# A Mobile App (Joint Effort) to Support Cannabis Use Self-Management and Reinforce the Use of Protective Behavioral Strategies: Development Process and Usability Testing

**DOI:** 10.2196/71924

**Published:** 2025-06-23

**Authors:** José Côté, Patricia Auger, Gabrielle Chicoine, Jinghui Cheng, Sylvie Cossette, Guillaume Fontaine, Christine Genest, Shalini Lal, Judith Lapierre, M Gabrielle Pagé, Marc-André Maheu-Cadotte, Geneviève Rouleau, Billy Vinette, Didier Jutras-Aswad

**Affiliations:** 1 Faculty of Nursing Université de Montréal Montreal, QC Canada; 2 Research Centre Centre Hospitalier de l’Université de Montréal Montreal, QC Canada; 3 Research Chair in Innovative Nursing Practices Montreal, QC Canada; 4 Knowledge Translation Program Li Ka Shing Knowledge Institute St. Michael's Hospital Toronto, ON Canada; 5 Department of Computer Engineering and Software Engineering Polytechnique Montréal Montreal, QC Canada; 6 Montreal Heart Institute Montreal, QC Canada; 7 Ingram School of Nursing Faculty of Medicine and Health Sciences McGill University Montreal, QC Canada; 8 Centre for Clinical Epidemiology Lady Davis Institute for Medical Research Jewish General Hospital Montreal, QC Canada; 9 Centre for Nursing Research Jewish General Hospital Montreal, QC Canada; 10 Viral Hepatitis Clinical Research Program Kirby Institute UNSW Sydney Sydney Australia; 11 Centre for Implementation Research Methodological and Implementation Research Program Ottawa Hospital Research Institute Ottawa, ON Canada; 12 Centre d’étude sur le trauma Research Centre Hôpital Louis-H Lafontaine Montreal, QC Canada; 13 School of Rehabilitation Faculty of Medicine Université de Montréal Montreal, QC Canada; 14 Douglas Research Centre Douglas Mental Health University Institute Montreal, QC Canada; 15 Faculty of Nursing Université Laval Québec, QC Canada; 16 Center for Research on Social Innovations Université Laval Quebec, QC Canada; 17 Departement of Anesthesioloy and Pain Medecine Faculty of Medecine Université de Montréal Montreal, QC Canada; 18 Department of Nursing Université du Québec en Outaouais Gatineau, QC Canada; 19 Institute for Health System Solutions and Virtual Care Women's College Hospital Toronto, ON Canada; 20 Institut du Savoir Montfort Ottawa, ON Canada; 21 Department of Psychiatry and Addictology Faculty of Medecine Université de Montréal Montreal, QC Canada

**Keywords:** cannabis, intervention, mobile app, mHealth, self-management, young adults, students, development, usability, prototype

## Abstract

**Background:**

Canada’s legalization of recreational cannabis use (CU) has further highlighted the need for innovative interventions that promote lower-risk CU. Young adults aged 18-25 years represent the age group with the highest prevalence of CU. Protective behavioral strategies (PBSs) have been shown to help manage CU and reduce its negative consequences. To date, only a few interventions have focused on PBSs. To address this gap, a mobile app prototype using PBSs to influence CU was developed with and for young adults.

**Objective:**

This study aims to describe the development process and usability testing of Joint Effort, a CU self-management mobile app prototype centered on promoting the use of PBSs among young adults with any past 30-day CU.

**Methods:**

Intervention mapping (IM) and a co-design approach were used. Six steps were followed: (1) focus groups were conducted to identify needs and preferences regarding CU interventions; (2) a matrix of change objectives was used to select target behaviors and determinants; (3) theory-based intervention methods and practical applications were selected; (4) focus groups were held to validate the intervention structure and examples of tailored messages; (5) preliminary intervention content was created; and (6) the intervention content was transposed into a mobile app prototype. Usability was assessed through qualitative semistructured interviews and the User Version of the Mobile Application Rating Scale (uMARS), completed by a sample of 20 university students with a mean age of 21.8 (median 22) years, 14 (70%) of whom were women and 15 (75%) were undergraduates. Qualitative data were analyzed using thematic analysis.

**Results:**

Four themes were identified from the interviews: Joint Effort was visually pleasing and easy to use; the content was well-adapted to the target audience and nonjudgmental; customization functions were appreciated; and the app was perceived as helpful and relevant for initiating behavior change. The prototype received a mean quality score of 4.43/5.0 (SD 0.53) per item on the uMARS. The mean scores on the 5 subscales were as follows: engagement (4.14, SD 0.53), functionality (4.60, SD 0.47), aesthetics (4.53, SD 0.52), information quality (4.44, SD 0.61), and subjective quality (3.36, SD 0.53).

**Conclusions:**

Our findings highlight the added value of IM and a co-design approach, underscoring the importance of incorporating user feedback in the development of mobile apps. Building on the strong usability results, the Joint Effort prototype has since been developed into an iOS mobile app, and larger-scale evaluations are currently underway to assess its acceptability, feasibility, and efficacy.

## Introduction

### Background

Previously conducted prospective cohort studies have suggested that substance use begins in adolescence and peaks in young adulthood [1], and that regular cannabis use (CU) typically starts between the ages of 18 and 19 years [2]. In Canada, the 18-24-year age group has been found to have the highest percentage of cannabis users [3].

Recreational CU was legalized nationwide in Canada in 2018 [4]. Since then, the use of cannabis products has increased considerably, particularly among the 18-25-year age group [1,5]. It has been shown that increased availability of legal cannabis can lead to higher CU and associated riskier use behaviors [6]. Legalization has made it all the more imperative to develop and offer preventive interventions aimed at reducing CU-related harm and encouraging safer behaviors [7]. Young adults, in particular, stand to benefit from such initiatives, given the high prevalence of CU in the 18-25-year age group and the limited number of existing interventions designed specifically for them [8].

A new generation of interventions targeting CU through digital technologies and mobile health (mHealth) has drawn researchers’ attention over the past decade. Several systematic reviews and meta-analyses [9-14] have demonstrated the relative effectiveness of various digital interventions in reducing CU frequency, although the effect sizes have been relatively small. However, these reviews have primarily focused on web-based interventions.

Smartphone usage and social media are increasingly popular among young people [15], and young adults are open to digital health services, especially when delivered via mobile apps [16]. However, our own systematic review of the literature found that none of the digital CU-related interventions available through mobile apps had been evaluated using a randomized controlled trial (RCT) [17]. A scoping review based on 5 studies, which aimed to explore the technical and functional characteristics of mHealth apps, found that this intervention modality demonstrated adequate feasibility and acceptability [18]. The following CU-related mobile apps were covered in the review: APPT (Assess, Plan, Track, and Tips; 18-50-year age group; Australia) [19]; MiSARA, a substance abuse research assistant (16-24-year age group; United States) [20]; VoltEgySzer (Once Upon a High; 14-18-year age group; Hungary) [21]; Stop-cannabis (14-59-year age group; Switzerland) [22]; and MApp (Marijuana Smartphone App; 18-25-year age group; United States) [23]. Since this review, 2 additional mobile apps have been mentioned in the scientific literature: HAP-app, a mobile app intended to help individuals reduce or cease CU (Norway) [24]; and LooseLeaf, a mobile app for young people at clinically high risk for psychosis, designed to help monitor CU and cannabis-related experiences (14-30-year age group; Canada) [25]. In short, the current research on CU mobile apps remains at an early stage, and most published studies have targeted either specific clinical populations or broad segments of the general population.

An innovative and positive way of promoting lower-risk CU is through the use of protective behavioral strategies (PBSs). PBSs are approaches that can help mediate CU and reduce negative CU consequences [26-28]. They involve behaviors—enacted immediately before, during, or after using cannabis—that support better CU self-management [26]. The use of PBSs has been associated with reduced negative effects of CU and lower CU frequency and quantity in a population of young university students [26-28]. In a previous study involving Canadian university students who reported any CU in the past 30 days (n=211), we found that greater use of PBSs was related to lower CU frequency, and that daily cannabis users employed fewer marijuana PBSs on average than lower-frequency users did [28]. A recent scoping review showed that PBSs may be associated with reduced CU-related negative consequences [29].

While the incorporation of PBSs is linked to CU self-management and holds great promise for lower-risk CU interventions, there has been very little research on PBS-centered interventions beyond the realm of alcohol use [9], and only a few interventions to date have targeted CU specifically [30]. Preliminary testing of MApp, a smartphone app used as part of a brief in-person intervention, indicated that PBS use reduced CU and that young adults were inclined to use a mobile app to help moderate their CU [23]. More recently, Lewis et al [31] published an RCT study protocol to evaluate a brief PBS-focused web-based and SMS intervention, although the results have yet to be published.

In sum, although the evaluation of PBS use as an intervention target in the context of safe CU is still at an early stage, findings to date suggest that PBS use is a positive and innovative approach to promoting lower-risk CU [31]. Against this background, we used intervention mapping (IM) to develop a mobile app prototype designed to support CU self-management and reinforce PBS use, with the goal of promoting lower-risk CU among young adults in the context of legalized recreational CU in Canada.

### Objective

This study aimed to develop Joint Effort, a mobile app prototype designed to support CU self-management and reinforce PBS use among young adults, and to assess its usability.

## Methods

### Structured Intervention Development Through IM

IM is a rigorous, theory- and evidence-based approach that emphasizes the role of research and theory in the intervention development process [32]. Our process was based on IM and a co-design approach. It included 6 steps: (1) conducting focus groups to identify the needs and preferences of young adults regarding CU interventions; (2) creating matrices of change objectives to select target behaviors and determinants; (3) selecting theory-based intervention methods and practical applications; (4) developing preliminary intervention content and structure; (5) conducting focus groups to validate the intervention structure and examples of tailored messages; and (6) transposing the intervention content into a mobile app prototype. The completion of one step guided the development of the next [32]. Various research phases and activities were embedded in the IM steps. These are described below.

Usability testing aimed to document the experience of using the mobile app prototype. This was conducted through semistructured interviews and the User Version of the Mobile Application Rating Scale (uMARS) [33]. A multimethod approach combining quantitative and qualitative data is generally recommended in usability testing to provide a more comprehensive understanding of user experiences [34]. The methods and results are reported in accordance with the Consolidated Criteria for Reporting Qualitative Research (COREQ) [35] (see Multimedia Appendix 1).

The target population for this study was selected based on 3 elements. First, substance use peaks in young adulthood [1], and the 18-24-year age group has the highest percentage of cannabis users [4]. Second, the legal age to purchase cannabis in Canada varies from 18 to 21 years, depending on provincial laws [36]. In Québec, where this study was conducted, it is illegal for anyone under 21 years to purchase cannabis. The lower age threshold for the study population was therefore set at 21 years. Third, most research on PBSs has been conducted with young adults and college or university students [26,37,38]. Given that the proof of concept for interventions based on PBSs has been demonstrated with this clientele, an academic setting was favored. This is why university students aged 21-24 years were chosen as a convenience sample to represent the young adult demographic. Recruitment was conducted at the Université de Montréal, a predominantly French-language university located in a metropolitan region (Montreal, Québec, Canada) in Eastern Canada.

### Development Process (IM Steps)

#### Step 1: Focus Groups to Identify Needs and Preferences

##### Overview

Focus groups were conducted to identify needs and preferences related to CU interventions.

##### Participant and Procedures

Participants were recruited through posters and social media advertisements on campus and in various Facebook student groups. We aimed to recruit at least 15 students aged 21-24 years from the Université de Montréal (Québec, Canada).

The 3 focus groups were conducted in person and led in French by 2 members of the research team. Two major topics were addressed: cannabis-related needs and interests, and preferences for technology-based interventions. These were explored through questions such as “What aspects of CU would you like to know more about?” “In your opinion, what constitutes safe and informed CU behaviors?” “How would you like to receive information or support (eg, device type, medium, form)?” “What might motivate you to use a digital intervention to receive information and support regarding your CU?”

All focus groups were audio-recorded and transcribed with participants’ consent. Each participant received CAD $50 (US $36.4) as compensation for their participation.

##### Analysis

Thematic analysis principles were used to analyze the data collected through the focus groups [39]. The transcripts were read multiple times to develop a deductive thematic coding tree. Three coders pilot-tested the coding tree by independently coding 1 of the focus groups. Differences among the coders were resolved through discussion, and the coding was then merged. The coding tree was finalized, and the definition of each code was discussed in depth before being applied to the remaining 2 transcripts. Descriptive codes were grouped into higher-order thematic categories, and the relationships between themes were detailed in a narrative summary. The summary was reviewed by the first author (JC). NVivo version 12 (QSR International Pty Ltd) was used for data management.

##### Findings

A total of 13 participants met the eligibility criteria (ie, university students aged 21-24 years who reported CU) and were divided into 3 groups of 4 or 5, based on their availability. The focus groups were conducted in December 2019 and January 2020. Sessions lasted 61, 62, and 76 minutes, respectively. Most participants self-identified as women (11/13, 85%). The mean age of participants was 22.4 (median 22) years.

Participants expressed that safe and responsible CU behaviors were characterized primarily by knowledge of the “facts” (eg, risks associated with CU, side effects of cannabis) or of “themselves” (eg, knowing one’s limits). Safe and responsible behavior involved ensuring that CU did not interfere with daily functioning or with personal, professional, and relational responsibilities. Participants emphasized the importance of having access to information and resources regarding CU.

In terms of technology preferences, participants emphasized that CU self-management interventions should be personalized and customizable. For example, they indicated that the amount and depth of information provided, notification frequency, and message format (eg, video, audio, or text) should be tailored to individual preferences. Additionally, participants suggested that the proposed intervention should be discreet, user-friendly, and engaging. They also expressed interest in having access to a CU monitoring feature (eg, where, when, quantity, with whom) to support self-monitoring and personal goal setting. The themes identified through thematic analysis, along with associated quotations, are presented in Multimedia Appendix 2.

##### Key Implications for Design

The data generated during the focus groups contributed to a deeper understanding of the needs and preferences of potential intervention users and laid the groundwork for the subsequent steps of intervention development. According to the target population, the intervention tool should be both educational and fun. It should provide information that is reliable, easily accessible and understandable, confidential, personalized, and encouraging. Additionally, it should include a CU logbook, reminders, and positive feedback.

#### Step 2: Matrix of Change Objectives

The process of creating a matrix of change objectives involved 3 steps: (1) specifying performance objectives (POs); (2) selecting important and changeable determinants of behavior; and (3) determining specific change objectives. The goal of the proposed intervention was to promote CU self-management and reinforce PBS use among young adult cannabis users. The target behaviors (ie, CU self-management and PBS use) encompassed various subbehaviors, which were translated into 4 POs described in Table 1.

**Table 1:** Matrix of change objectives.

Performance objectives	Determinants			
	Attitude	Self-efficacy	Social norms	Intention
Gain awareness of your CU^a^ (frequency and consequences).	Assess the reasons for wanting to change your CU.	Identify risky situations related to your CU.	Determine how you measure up against other users.	N/A^b^
Set a goal for yourself.	Appreciate the importance of setting this goal.	Reflect upon your capacity to pursue this goal.	N/A	Reflect upon and assess your motivation and intention for setting this goal.
Commit to a change process and strive to achieve your goal.	Identify the benefits of change.	Identify possible obstacles and facilitators. Focus on means and resources.	N/A	Formulate an action plan based on PBS^c^.
	Focus on the benefits of and motivation for change.	Identify ways of overcoming obstacles.	N/A	Implement your action plan.
Overcome possible obstacles and consolidate your goal.	Remind yourself of the benefits identified.	Call on factors capable of facilitating change and on external resources.	N/A	Revise or adjust your action plan.

^a^CU: cannabis use.

^b^N/A: not applicable.

^c^PBS: protective behavioral strategy.

Considering the explanatory power of the Ajzen Theory of Planned Behavior (TPB) in behavior change and adoption [40], 4 TPB determinants were targeted to promote CU self-management and PBS use, namely, attitude, perceived behavioral control (self-efficacy), social norms, and intention [40]. In the context of CU, a few studies have shown that higher levels of self-efficacy are significantly associated with increased PBS use [41,42]. Attitude and perceived behavioral control/self-efficacy have also been found to predict the behavioral intention to use PBSs [43].

The intersection of POs and specific behavioral determinants to be targeted is presented in Table 1. The specific change objectives outline what an individual needs to do to optimize CU self-management and PBS use.

#### Step 3: Selection of Theory-Based Intervention Methods and Practical Applications

In IM, it is crucial to select theory-based intervention methods and practical applications—referred to as behavior change techniques (BCTs) in other approaches [44]—that appropriately align with the targeted determinants [32].

To support this process, a thorough BCT analysis was conducted to identify explicit behavior change mechanisms reported in digital interventions for recreational CU among young adults [17]. The most frequent BCT clusters identified were “Feedback and monitoring,” “Goals and planning,” “Natural consequences,” and “Comparison of outcomes.” Feedback on behavior emerged as a core component in nearly all of the CU behavioral interventions analyzed. These findings provided valuable insights for identifying the key active ingredients necessary to develop an effective intervention.

Specific theory-based intervention methods were selected to guide practical applications that effectively address the targeted behavioral determinants and support behavior adoption (ie, PBS use). These methods included personalized feedback, modeling, verbal persuasion, self-monitoring, and positive reinforcement. Examples of these methods and their corresponding practical applications are presented in Table 2.

**Table 2:** Examples of theory-based intervention methods and their practical applications used to target determinants.

Targeted determinants and theory-based intervention method			Examples of practical applications
**Intention**			
	Goal setting	SMART^a^ action plan	
	Activation of intention	If/then technique	
**Self-efficacy**			
	Modeling	Lived experience: CU^b^ consequences	
	Coping planning	Identify obstacles and ways to overcome them	
**Attitude**			
	Belief selection	Reflective questions regarding motivation to change	
	Anticipated outcome	Benefits of adopting a new behavior	
**Social norms**			
	Personalized feedback	Feedback regarding CU frequency	

^a^SMART: Specific, Measurable, Achievable, Relevant, and Time-bound.

^b^CU: cannabis use.

Integrating results from the initial steps of the IM process led to the development of a logic model of change that illustrates the underlying mechanism. Based on the target behavior and subbehaviors, the determinants and theory-based intervention methods form the active ingredients of the mobile app designed to increase the use of PBSs (principal outcome). Increased PBS use is expected to be associated with a decrease in the frequency of CU (secondary outcome). The determinants (intention and self-efficacy) serve as mediating variables in this model, as illustrated in Multimedia Appendix 3.

#### Step 4: Creation of Preliminary Intervention Content and Structure

The preliminary structure and content of the intervention were developed based on the matrix of change objectives established in step 2 and the theory-based intervention methods selected in step 3. Subgroups of the research team, along with a working group of potential young adult users, were formed and met multiple times to cocreate the intervention content.

The title of the intervention—Joint Effort—relies on a playful pun (which also works in French) and suggests that users will need to put in some work to change, though they will be supported in doing so.

As detailed in Table 3, the content was presented in 5 sections: (1) Assess—gain awareness of your CU; (2) Mobilize—support your decision to take action; (3) Act—support the establishment of your action plan; (4) Strengthen—consolidate change (booster session); and (5) Observe—monitor your CU.

The intervention was designed to be self-directed and, as such, focused on simple strategies that users could apply independently without relying on external resources.

**Table 3:** Summary of intervention content.

Section	Aim	Format/components/features	Topics/key content
Assess	Enable users to gain a better awareness of their CU^a^	Questions and personalized feedback	CU frequency, CU motivation, CU consequences, and motivation to change
Mobilize	Support decision-making process	Generic/general messages and questions to reflect upon	PBSs^b^, benefits of change, possible difficult situations, and strategies and resources
Act	Support the establishment of an action plan	Generic/general messages, questions (for the SMART^c^ action plan), personalized feedback, and questions to reflect upon	SMART action plan, anticipate difficulties and barriers (if/then), and how to overcome obstacles
Strengthen	Support the sustainability of the action plan	Personalized feedback	The SMART action plan (booster)
Observe	Personalized monitoring	Questions and personalized feedback	CU logbook (eg, daily CU frequency, products, open text log) and SMART action plan follow-up

^a^CU: cannabis use.

^b^PBS: protective behavioral strategy.

^c^SMART: Specific, Measurable, Achievable, Relevant, and Time-bound.

#### Step 5: Focus Groups to Validate Intervention Structure and Examples of Tailored Messages

##### Overview

Focus groups were conducted to validate the mobile app plan and examples of personalized messages used to develop the prototype.

##### Participants and Procedures

All participants from the previous focus groups (step 1; n=13) were invited to take part in this validation phase, and 8 agreed (4 were unavailable and 1 did not respond). Three new participants were recruited through ads posted in various Facebook groups targeting students, as well as via snowball sampling. All of these new focus groups were conducted online via Zoom (Zoom Communications), in French, by the same team as in step 1.

Participants discovered the intervention content during the focus group through a PowerPoint (Microsoft Corporation) presentation that summarized the structure and included examples of messages. Questions such as “What do you think of the topics covered?” “What do you think of the sample message?” and “How could it be improved?” were used to lead the discussion.

All meetings were recorded and transcribed, with field notes taken. Participants received CAD $50 (US $36.4) for taking part in the focus groups.

##### Analysis

The transcripts of the meetings and field notes were summarized. Comments and suggestions were organized thematically. The research team then discussed the results to agree on any changes to be made.

##### Findings

In December 2020, 11 participants were divided into 4 groups of 2 or 3 based on their availability, and 1 participant was interviewed individually. The mean age was 22.2 (median 22) years, with 7 out of 11 (64%) participants being women. The focus groups lasted between 49 and 115 minutes (mean 83.3 minutes; median 84.5 minutes), while the individual interview lasted 28 minutes. Duration was mainly influenced by the number of participants.

Regarding the main objective, participants emphasized that not all cannabis users may be interested in reducing or modifying their use; some might only want to track it. In this regard, they highlighted the importance of having a logbook option available to all users at all times (ie, not dependent on the completion of previous sections).

Overall, the proposed structure and sequence (ie, 5 sections: assess, mobilize, act, strengthen, and observe) were received positively. Participants appreciated the topics and progression of content, the personalized feedback, and the intervention’s name—Joint Effort.

Moreover, the examples of messages were considered interesting and appropriate. Participants indicated that personalized feedback could help reassure them and normalize their behavior. For example, personalized feedback in the Assess section provided information about CU frequency and motivation based on the results of a population-based survey [45]. For instance, to the question “In the past month, how often have you used cannabis?,” users could receive the following feedback “Frequency: regular. 38% of Quebecers aged 18-24 report having used cannabis in the past year. Among those who use it, just like you, 1 out of 5 did it regularly”. To the question “Thinking back over the past month, for what reason(s) have you used cannabis?,” they could receive the following message “Like you, 9 out of 10 people who regularly use cannabis take it for fun” or “Like you, 96% of people who regularly use cannabis take it to relax”.

The proposed strategies (ie, PBSs) were considered relevant, and the list of examples was appreciated. However, the vocabulary and phrasing received mixed reviews. In some cases, the text felt overly formal and disconnected from the reality experienced by young people.

The themes that emerged from the analysis, along with associated quotations, are presented in Multimedia Appendix 4.

##### Key Implications for Design

Validating the content and message algorithms was an essential step in the design process before prototyping the mobile app. Participants appreciated the proposed intervention structure, its conciseness and comprehensiveness, as well as the personalized messages, visual feedback, and graphic representations.

Minor changes were made to the intervention structure. For example, the “Observe” section (ie, CU logbook) was initially accessible only after completing the “Assess” section. Based on feedback from focus groups—highlighting that some users might be interested solely in tracking their CU—it was decided to make this section accessible from the start, allowing users to access their logbook without completing any prerequisite sections. Additionally, users could complete the first 3 sections (Assess, Mobilize, and Act) at their convenience; an order of completion was suggested but not imposed.

All intervention content was reviewed to better tailor the examples and language to the target population. A committee representing potential end users, composed of 4 focus group participants, was formed to help fine-tune the intervention. They revised and approved all updated texts and messages in the final validation loop.

#### Step 6: Transposing the Intervention Content to a Mobile App Prototype

##### Overview

The intervention content was transposed into a mobile app format. At this stage of the iterative prototype development process, members of the potential end users committee (formed in step 4) were actively involved in validating the prototype.

##### Intervention Content Scripting and Wireframes

The intervention content validated in step 4 was scripted into short messages organized to create a navigation algorithm (user flow). This content included general information messages, personalized feedback, and questionnaires (checkbox answers and reflection questions) covering various topics. It also featured interactive components, such as a self-monitoring function to track CU. More than 100 wireframes were created using InVision (Dribbble Holdings Ltd) and validated.

##### Graphic and Visual Design

**Figure 1:**
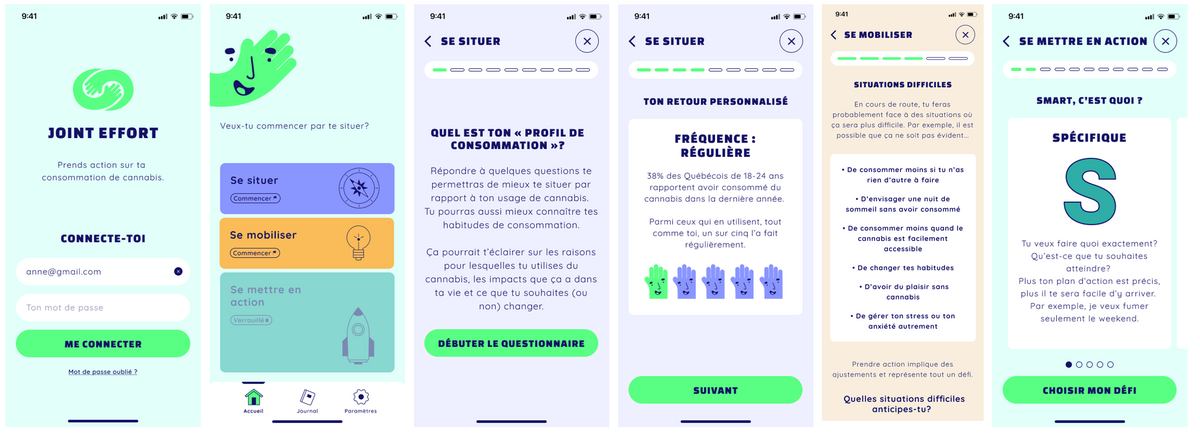
Selected screenshots of the Joint Effort mobile app prototype.

The graphic identity was designed to appeal to the target audience. Several iterations of the chosen logo and screen mock-ups were produced and validated by the potential end user committee to finalize the graphic identity. Over 50 illustrations were created to support the messages conveyed in the theoretical content of the intervention. Examples of screenshots from the Joint Effort prototype are presented in Figure 1.

Elements of gamification were integrated into the prototype. A virtual character was created to be at the heart of the user experience, guiding users through various app steps (tutorial, transitions, home page, and logbook). The menu featured locked/unlocked sections to create a playful visual dynamic, and a personalized dashboard allowed users to visualize their progress and see how much of their goal had been achieved.

The user interface was designed with a focus on the user journey. The prototype was created using Figma (Figma, Inc), a collaborative design and prototyping tool [46].

##### Planned Exposure

In terms of dosage, the intervention was intended to be visited multiple times over a 30-day period. Users could view the content at their own pace (eg, 1 session per day or more than 1 per day). However, they had to wait 7 days before accessing the Booster section.

In terms of duration, the estimated time to complete all the content (ie, Assess, Mobilize, Act, and Strengthen) was about 15 minutes. The time devoted to the Observe section (ie, CU logbook) varied depending on the amount of data input (ie, up to each individual).

### Usability Testing

#### Participant Selection and Sample Size

The inclusion criteria for participants were being a university student at Université de Montréal, being 21-24 years old, and having engaged in any CU in the past 30 days. Recruitment ads were posted in various Facebook groups targeting students. Interested individuals contacted the study team and had their eligibility confirmed. A sample size of 20-30 participants was targeted, as proposed by Thabane et al [47].

#### Data Collection

##### Think-Aloud Interviews

As suggested by Noushad et al [48], we purposefully reflected on the context and research questions before choosing to use the think-aloud method. This method was selected to capture real-time cognitive processes and insights of participants as they performed a specific task (ie, using the mobile prototype). The methodology focuses on asking participants to verbalize their thoughts while completing a task or to recall what was going through their minds after performing it [49]. At that phase, the app was available only as a Figma prototype (ie, accessed via a private URL, with limited features and navigation that needed to be explained). These interviews aimed to understand users’ spontaneous reactions to the intervention content, to document how they interacted with the components, and ultimately to inform usability testing.

The interviews were conducted online (via Zoom), in French, by 2 members of the research team (same as in step 1 and 5) with prior experience in qualitative research. Participants were invited to use a prototype of the intervention (via Figma) while thinking out loud about various aspects, including aesthetics, navigability, and reactions to the content. Using a shared link in a web browser, they viewed mock-ups of an iPhone screen featuring visual elements (images and text) and interactive buttons simulating the mobile app.

No interview guide was used. Instead, interviewers encouraged participants to verbalize what they saw, thought, and did while interacting with the prototype. Reformulation and clarification questions (eg, “You mentioned that..., can you tell me more about it?”) were asked for explanatory purposes. All interviews were audio-recorded and transcribed.

After each interview, participants were invited to complete a short online sociodemographic questionnaire and the uMARS [33]. Unique links to the questionnaires were sent via LimeSurvey (LimeSurvey Team; Université de Montréal license).

The short sociodemographic questionnaire aimed to collect information to describe the study sample (eg, gender, age, educational level, CU frequency).

The uMARS is a reliable tool for assessing the quality of mHealth apps. It consists of 20 items grouped into 4 quality subscales—engagement, functionality, aesthetics, and information—as well as 1 subjective quality subscale. Each item is rated on a 5-point scale ranging from 1 (inadequate) to 5 (excellent). According to Stoyanov et al [33], the uMARS has demonstrated excellent internal consistency for the full scale (Cronbach α=0.90). To determine an app’s quality score on the uMARS, Stoyanov et al [33] suggested calculating the mean score for each of the 4 quality subscales and then averaging these 4 mean scores. The uMARS was translated from English to French following a rigorous process similar to that used for the 17-item version of the Protective Behavioral Strategies for Marijuana Scale [28].

Participants were offered CAD $30 (US $21.8) for taking part in the usability testing.

##### Data Analysis

The data analysis process for usability testing was the same as that presented in step 1. Descriptive statistics were used to analyze the quantitative data, with Excel software (Microsoft Corporation) used for this purpose.

### Ethical Considerations

All steps of the study were approved by the Centre Hospitalier de l'Université de Montréal Research Ethics Board (approval number 20.172) and the Comité d’éthique de la recherche en sciences et en santé (CERSES) of the Université de Montréal (approval number CERSES-20-114-D). All participants provided informed consent. All participants provided informed consent by signing a copy of the informed consent form. Compensation to participants was disclosed in previous sections. Privacy and confidentiality of participants’ data or identity were maintained during the study process and publication.

## Results

### Sociodemographic Characteristics

In May 2021, usability testing was conducted with 20 participants whose mean age was 21.8 (median 22) years. Most were women (14/20, 70%), born in Canada (15/20, 75%), and undergraduates (15/20, 75%). Additionally, most reported engaging in weekly CU in the past 30 days (14/20, 70%).

### Think-Aloud Interviews

#### Overview

A total of 20 think-aloud interviews were conducted, lasting between 20 and 51 minutes. Four themes emerged from the interviews: (1) the mobile app prototype was visually pleasing and easy to use; (2) the content was well adapted to the target audience and nonjudgmental; (3) the customization possibilities were an appreciated feature; and (4) the mobile app was helpful and relevant for initiating behavior change. Examples of participant verbatim quotations are presented in Table 4.

**Table 4:** Themes and associated quotes (think-aloud analysis).

Themes	Examples of quotes^a^
Visually pleasing and easy to use	“The colors are nice, soft, pastel.” [ID01] “I personally like the little images, the little drawings, a lot because I find that it brings in a playful side that’s not too serious.” [ID14] “For real, I think the app is easy to use, and the information on it is clear and precise.” [ID04]
Content well adapted to target audience and nonjudgmental	“What I liked best was that when I used this app I didn’t feel like I was being judged....” [ID10] “I think it’s a good thing that right from the start the intentions are announced, we see that it’s really meant to help to take action.” [ID11] “Yes, yes, there are lots of choices, I think that it could represent a fair portion of users.” [ID20] “Yeah, I like that it gives me little facts as well, I find that it normalizes consumption, I don’t feel like stigmatized.” [ID10]
Customization possibilities, an appreciated feature	“I like it because so far it really leaves it up to the consumer....You go at it how you feel, the app is there but you go at it at your pace.” [ID14] “The option of being able to go through it all at once or in several parts is good.” [ID19]
Relevant to initiate behavior change	“It allows you to question your own consumption, put it back into perspective, understand why you use, if ever you wanted to change something.” [ID01] “OK, so, strategies before, you see I’ve been smoking for a really long time, and I’ve never seen any strategies before....It’s really more informative and educational.” [ID08] “The mobile app is already a good step forward and it’s a good compromise and it’s easy to access.” [ID15] “I think that it can be just as useful to someone who’s just trying to stop though it’s more to gain some insight on the bad effects of your consumption...even if you don’t necessarily stop completely, just understanding and knowing your use, it’s all good.” [ID3]

^a^Quotes were translated from French to English by a specialized translator and validated for accuracy by the research team.

#### Visually Pleasing and Easy to Use

Participants expressed positive feedback regarding the app’s visuals. The visual layout was considered uncluttered, and the neutral, soft, and soothing color scheme was appreciated. The images and virtual characters were seen as playful and amusing without being childish. Participants found the app easy to use, clear, and accessible. Navigation was described as fluid and intuitive, with participants finding it easy to find their way around.

#### Well-Adapted to Target Audience and Nonjudgmental

The neutral tone and nonjudgmental wording contributed to a nonmoralizing aspect of the app. As the app’s goals were clearly stated in the introduction (ie, “aims to help you take action on your cannabis use, not to convince you to stop or change at any cost”), participants felt reassured about its purpose. In the personalized feedback, the comprehensive statistics helped normalize CU without stigmatizing it. When selecting answers from a set list, participants felt the choices reflected their reality and were well adapted to the target audience.

#### Customization Possibilities, an Appreciated Feature

The various customization aspects of the app were highly appreciated. For instance, the option to choose or write personalized goals was particularly appealing to some participants. They also liked the flexibility of going through the content at their own pace, as they could either complete most sections in a single sitting or pause and return to them later if they wished.

#### Relevant to Initiate Behavior Change

Participants highlighted various benefits of the app for all types of users. It offered support and monitoring for those wishing to initiate a process of behavior change. The information and strategies presented in the app were considered relevant and educational, and were seen as encouraging users to reflect on their CU. Participants also noted that the app could help facilitate access to other services.

### uMARS

After the think-aloud portion of the interview, participants (n=20) were sent a link to complete the uMARS. Results per item and per subscale are presented in Table 5. The Joint Effort prototype received the following mean scores on the 4 subscales: 4.14/5.0 (SD 0.53) for Engagement, 4.60/5.0 (SD 0.47) for Functionality, 4.53/5.0 (SD 0.52) for Aesthetics, and 4.44/5.0 (SD 0.61) for Information. This yielded an overall app quality mean score of 4.43/5.0 (SD 0.53). The mean scores on the subjective items varied widely. The item “Would you recommend this app to people who might benefit from it?” received the highest score at 4.35/5.0 (SD 0.67), while “Would you pay for this app?” received the lowest at 1.65/5.0 (SD 1.09).

**Table 5:** Results on the User Version of the Mobile Application Rating Scale (N=20).

Subscale and item			Mean (SD)		Median (range^a^)
**Engagement**			4.14 (0.53)		N/A^b^
	1. Entertainment: Is the app fun/entertaining to use? Does it have components that make it more fun than other similar apps?	4.05 (0.51)		4 (3-5)	
	2. Interest: Is the app interesting to use? Does it present its information in an interesting way compared to other similar apps?	4.60 (0.50)		5 (4-5)	
	3. Customization: Does it allow you to customize the settings and preferences that you would like to (eg, sound, content, and notifications)?	3.80 (0.89)		4 (2-5)	
	4. Interactivity: Does it allow user input, provide feedback, and contain prompts (reminders, sharing options, notifications, etc)?	3.65 (1.09)		4 (1-5)	
	5. Target group: Is the app content (visuals, language, and design) appropriate for the target audience?	4.60 (0.68)		5 (3-5)	
**Functionality**			4.60 (0.47)		N/A
	6. Performance^c^: How accurately/fast do the app features (functions) and components (buttons/menus) work?	N/A		N/A	
	7. Ease of use: How easy is it to learn how to use the app? How clear are the menu labels, icons, and instructions?	4.75 (0.55)		5 (3-5)	
	8. Navigation: Does moving between screens make sense? Does the app have all the necessary links between screens?	4.60 (0.50)		5 (4-5)	
	9. Gestural design: Do taps/swipes/pinches/scrolls make sense? Are they consistent across all components/screens?	4.45 (0.69)		5 (3-5)	
**Aesthetics**			4.53 (0.52)		N/A
	10. Layout: Is the arrangement and size of buttons, icons, menus, and content on the screen appropriate?	4.65 (0.67)		5 (3-5)	
	11. Graphics: How high is the quality/resolution of graphics used for buttons, icons, menus, and content?	4.70 (0.57)		5 (3-5)	
	12. Visual appeal: How good does the app look?	4.25 (0.64)		4 (3-5)	
**Information**			4.44 (0.61)		N/A
	13. Quality of information: Is app content correct, well written, and relevant to the goal/topic of the app?	4.50 (0.69)		5 (3-5)	
	14. Quantity of information: Is the information within the app comprehensive but concise?	4.20 (0.95)		4 (1-5)	
	15. Visual information: Is visual explanation of concepts—through charts/graphs/images/videos, etc—clear, logical, and correct?	4.70 (0.47)		5 (4-5)	
	16. Credibility of source: Does the information within the app seem to come from a credible source?	4.35 (0.93)		5 (2-5)	
**Subjective items**			3.36 (0.53)		N/A
	17. Would you recommend this app to people who might benefit from it?^d^	4.35 (0.67)		4 (3-5)	
	18. How many times do you think you would use this app in the next 12 months if it was relevant to you?^e^	3.25 (1.02)		3 (1-5)	
	19. Would you pay for this app?^f^	1.65 (1.09)		1 (1-4)	
	20. What is your overall (star) rating of the app?^g^	4.20 (0.41)		4 (4-5)	

^a^Possible score range: 1-5.

^b^N/A: not applicable.

^c^The item “Performance” (How accurately/fast do the app features [functions] and components [buttons/menus] work?) could not be evaluated on the Figma prototype.

^d^Choices of answer ranging from 1 “not at all” to 5 “definitely.”

^e^Choices of answer: 1=“none”; 2=“1-2”; 3=“3-10”; 4=“10-50”; 5=“>50.”

^f^Choices of answer ranging from 1=“definitely not” to 5=“definitely yes.”

^g^Choices of answer ranging from 1=“1 star” to 5=“5 stars.”

## Discussion

### Principal Findings

The objective of this study was to codevelop and conduct usability testing of a mobile app prototype, aimed at promoting CU self-management and reinforcing PBS use among university students.

Focus groups were conducted to identify the needs and preferences of young adults regarding CU intervention. The data collected during this phase informed the development of the intervention content. A second round of focus groups was then held to validate the structure of the intervention and examples of tailored messages it might convey. Subsequently, the intervention content was transposed into digital form through iterative software development, and the mobile app prototype was created. A third validation phase was carried out using think-aloud interviews and a short questionnaire to document the user experience.

Usability findings suggest that participants appreciated the app. The prototype received an overall app quality score of 4.43/5. The mean scores on the uMARS subscales were relatively high for Engagement (4.14/5), Functionality (4.60/5), Information (4.44/5), and Aesthetics (4.53/5). Interestingly, the item that received the lowest rating was “Would you pay for this app?” (1.65/5.0, SD 1.09). This suggests that, despite the app being perceived positively, participants were not willing to pay a subscription fee. A systematic review has shown that offering health-related apps for free or at a low cost can positively influence their uptake and engagement [50].

The results of the think-aloud interviews (qualitative component) aligned with the uMARS findings. Participants found navigation smooth and intuitive, which made it easy for them to find their way around, and the content conducive to prompting behavior change. A systematic review and thematic synthesis (n=35 studies) of mHealth interventions identified reinforcement, communication, navigation, credibility, message presentation, and interface aesthetics as key design features to consider for improving user engagement [51]. Given the strong uMARS scores obtained for these components, it is reasonable to expect that the Joint Effort app may successfully elicit user engagement.

However, comparing our uMARS results with those of other studies is challenging. Although this tool has been used elsewhere, most mobile apps evaluated with the uMARS differ substantially from Joint Effort. To date, in the field of cannabis and other substance use research, the use of uMARS has primarily been mentioned in protocols [52-54]. Only Santesteban-Echarri et al [55] reported using the uMARS to evaluate a cannabis-related mobile app for youth at high risk for psychosis. They found a good overall score (3.75/5) and high subscale ratings for esthetics (4.48/5), information (4.32/5), and functionality (4.29/5) [55]. In comparison, the Joint Effort prototype received a higher overall app quality score (4.43/5). The slightly higher scores obtained in our study may be attributed to the IM and co-design approach used, along with multiple validation rounds. The target population was involved from the outset (ie, needs analysis), and potential end users were engaged throughout the process. However, these comparisons should be interpreted with caution, as subjectivity is inherent when using a scale such as the uMARS. For instance, aesthetic preferences may vary across cultural groups. Moreover, the uMARS was completed after a single use of the app prototype, rather than after extended use of its final version.

The aim of the mobile app prototype developed is to promote PBS use, which involves setting a goal, engaging in a change process, and implementing an action plan (SMART, take action). Our qualitative results suggest that participants appreciated the opportunity to engage at their own pace, set their own goals, and initiate the behavior change they desired, if any. In a rapid review of the literature (n=43 studies), Monarque et al [56] concluded that the uptake of digital interventions among youth depended on the incorporation of harm reduction principles and skills training. These approaches were favored in the development of Joint Effort, as participants were supported in self-managing their CU by reinforcing PBS use, rather than being encouraged to quit altogether.

Different approaches were used to support CU self-management and reinforce PBS use in developing the app, including personalized feedback, anticipated outcomes, positive reinforcement, goal setting, self-observation, and activation of intention. In a systematic review examining the efficacy of behavior change smartphone apps (n=27 studies), Schoeppe et al [57] emphasized common strategies frequently used in successful app-based interventions, such as goal setting, self-monitoring, and performance feedback. These findings were later reinforced in the systematic review by Milne-Ives et al [58], which identified 6 common behavior change techniques associated with user engagement in mHealth apps: goal setting, self-monitoring of behavior, feedback on behavior, prompts/cues, rewards, and social support. In light of the above, the Joint Effort mobile app prototype was designed using the most effective strategies to promote engagement and intervention success. These strategies will be evaluated in subsequent research phases, with a focus on both experiential and behavioral engagement [59].

An unexpected element emerged from the qualitative interviews regarding the nonjudgmental approach used. Participants reported not feeling judged or stigmatized and expressed that their behavior felt normalized. This is particularly important given the ongoing stigmatization of CU, both in jurisdictions where cannabis is legal and where it remains illegal [4,60]. In terms of intervention access, several barriers have been documented, including limited availability, confidentiality concerns, and stigma [61]. The intervention was developed with the intention of serving as a tool for self-directed prevention, offering simple and achievable strategies that require no reliance on external resources. This could address a specific need among young adults who use cannabis and are interested in changing their consumption habits. The advantages of interventions delivered via mobile apps are well documented in the literature. For example, mobile apps provide an economical and easily accessible means of delivering low-intensity interventions for mental health–related issues [62]. Anonymity, asynchrony, and easy, immediate access (without the need to leave home) were the advantages most appreciated by participants in this study.

### Strengths and Limitations

The principal strength of this study lies in the rigorous, theory-based process used to cocreate the mobile app prototype. IM is a robust framework for developing theory- and evidence-based interventions [32]. Multiple methods were used to conduct an extensive needs analysis, including a systematic review [17], an online CU study [28,63], and focus groups. The overall IM process involved the continuous integration and analysis of 3 types of knowledge: empirical (previous studies and needs analysis), experiential (input from key informants), and theoretical (behavioral determinants). The iterative codevelopment process included multiple phases of consultation with potential end users of the mobile app and several validation loops to ensure the intervention aligned with user preferences and needs. Finally, the study was conducted by a well-rounded, thoughtfully assembled interdisciplinary team of researchers with diverse expertise, including cannabis, information and communication technologies, health promotion, youth mental health, knowledge transfer, and mixed methods research (both quantitative and qualitative), working in collaboration with potential end users.

A key limitation of the study is the overwhelmingly positive user reactions and reviews received during the think-aloud interviews. Participants did not mention any aspects they disliked or suggested elements for improvement at that time. This may be attributed to the methodology used: given the stage of the design process, participants interacted with the prototype for the first and only time during the interview. As such, the results reflected their initial general impressions. A more extended and in-depth use over time may have elicited more nuanced feedback.

Another limitation is the predominance of female participants across the focus groups conducted to identify user needs (11/13, 85%), validate the intervention content (7/11, 64%), and during the usability testing phase (14/20, 70%). Given that negative CU consequences are disproportionately concentrated in the young adult male population [7,29], it is reasonable to assume that a higher proportion of males with different viewpoints might have led to a slightly different intervention. In sum, these findings highlight the challenges of developing effective mobile app interventions for CU that account for gender differences across various clinical correlates (eg, readiness to change, sources of motivation for behavior change) that may influence young adults in their decision to take steps toward better managing their CU.

### Conclusions

In the context of the recent legalization of cannabis and its widespread consumption, strategies to promote safer use and reduce harm need to extend beyond traditional abstinence-based approaches. mHealth interventions appear promising in this regard, particularly in addressing challenges related to health care access and stigmatization. Following this study, and given the promising results from usability testing, we proceeded with the further development of the Joint Effort mobile app. While the work described in this manuscript yielded positive signals from potential end users, further evaluation is needed to confirm its acceptability, feasibility, and ultimately, its efficacy in terms of CU outcomes. A pilot randomized trial [64] was conducted to assess the intervention’s acceptability (user uptake, user engagement, user-participant profiles, and intervention appreciation) and to document the feasibility of the study process (online recruitment rate, adherence to online data collection methods, and attrition rate). Based on the logic model of change (Multimedia Appendix 3), an RCT is ongoing to evaluate the intervention’s efficacy [65] on PBSs as the primary outcome, with frequency of CU as a secondary outcome, and intention to take action on CU as a mediator outcome. If proven efficacious, Joint Effort could help diversify the available tools to improve CU outcomes.

## Appendix

Multimedia Appendix 1 COREQ checklist.

Multimedia Appendix 2 Results from focus groups to identify needs and preferences (step 1).

Multimedia Appendix 3 The logic model of change (step 3).

Multimedia Appendix 4 Results from focus groups to validate intervention structure and examples of tailored messages (step 5).

## Abbreviations

APPT: Assess, Plan, Track, and Tips
CERSES: Comité d’éthique de la recherche en sciences et en santé
COREQ: Consolidated Criteria for Reporting Qualitative Research
CU: cannabis use
IM: intervention mapping
MApp: Marijuana Smartphone App
mHealth: mobile health
PBS: protective behavioral strategy
PO: performance objective
RCT: randomized controlled trial
TPB: Theory of Planned Behavior
uMARS: User Version of the Mobile Application Rating Scale
