# Supplementary material for: A Mobile App (Joint Effort) to Support Cannabis Use Self-Management and Reinforce the Use of Protective Behavioral Strategies: Development Process and Usability Testing
Source: JMIR Form Res. 2025 Jun 23;9:e71924. doi: 10.2196/71924 (PMC12235202; doi:10.2196/71924)
Supplement: Multimedia Appendix 2 [file formative_v9i1e71924_app2.pdf]

Results from focus groups to identify needs and preferences (step 1)

Themes	Examples
Access to information and resources related to CU	*"Me, I'd say knowledge for sure (...) not just knowing more about the substance, the facts, how much, what types (...) but also getting to know yourself better, how much you can take, your experiences, your weaknesses, whatever they may be. So, for sure, I'd say that the key word is knowledge."(FG3)*<br>*"Information that is accessible, quickly and easily as well. You want it to be easy, you don't need to search for it."(FG2)*
Technology preferences	*"There are people who will like to receive a hundred notifications a minute. But there are others who will prefer like two a day (...) The app should have certain options and allow users a certain freedom to manage them so that you can adapt it to your needs (...) I keep coming back to the self, but it's really important (...) something that will really adjust to each person's reality." (FG1)*<br>*"We're always more inclined to use services when we have the impression that they were developed for us." (FG3)*<br>*"You need to strike the right balance between confidentiality and customization." (FG2)*<br>*"Sometimes, also, you might feel like opening the app in the subway but you don't feel like having anyone else looking over your shoulder (...) you want to keep it confidential so you don't want it to be flashy and draw attention (…) that it really be something rather discreet deep down." (FG1)*<br>*"What I would like is that the app have a logbook, that you could keep like a consumption logbook, and to note in it how you're feeling. […] that pushes you to reflect upon your consumption, but really without it being moralistic, just push you to reflect upon your own consumption."(FG1)*
