# Supplementary figures and images for: A Mobile App (Joint Effort) to Support Cannabis Use Self-Management and Reinforce the Use of Protective Behavioral Strategies: Development Process and Usability Testing
Source: JMIR Form Res. 2025 Jun 23;9:e71924. doi: 10.2196/71924 (PMC12235202; doi:10.2196/71924)

Logic Model of Change (step 3)

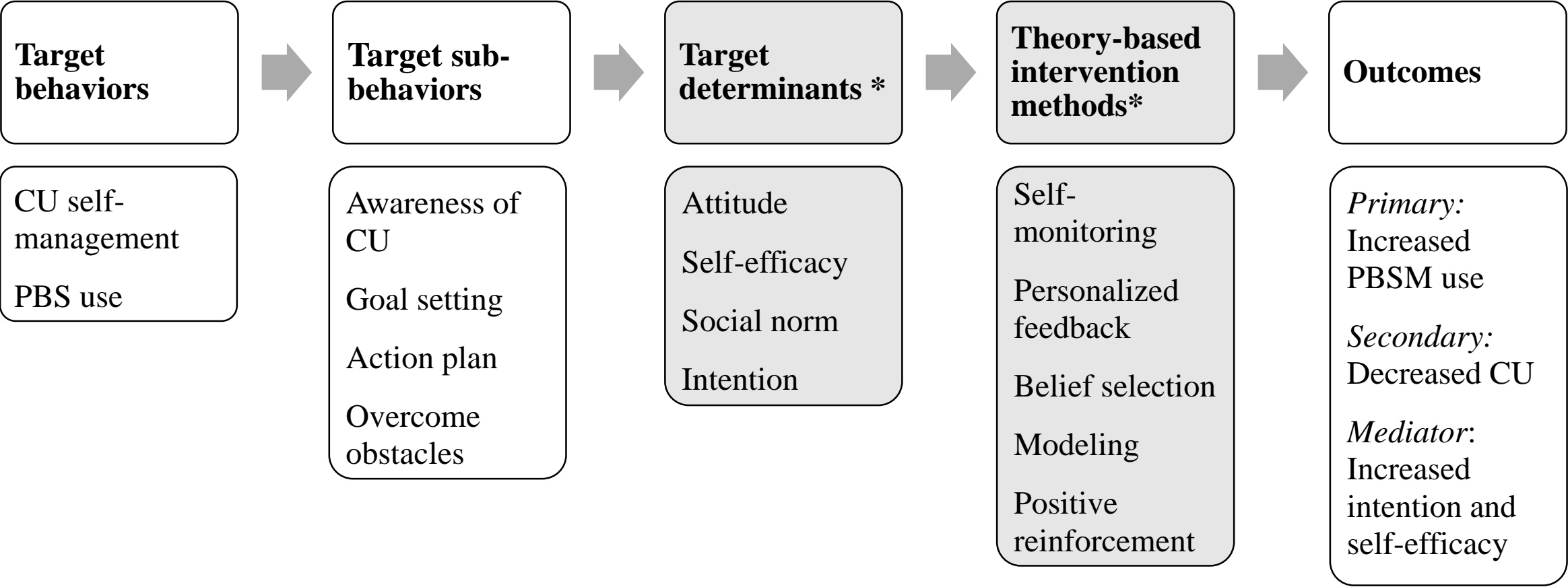

\* Active ingredients

Supplement: Multimedia Appendix 3 [file formative_v9i1e71924_app3.pdf]
