# Supplementary material for: A Mobile App (Joint Effort) to Support Cannabis Use Self-Management and Reinforce the Use of Protective Behavioral Strategies: Development Process and Usability Testing
Source: JMIR Form Res. 2025 Jun 23;9:e71924. doi: 10.2196/71924 (PMC12235202; doi:10.2196/71924)
Supplement: Multimedia Appendix 4 [file formative_v9i1e71924_app4.pdf]

Results from focus groups to validate intervention structure and examples of tailored messages (step 5)

Themes	Examples
Not all cannabis users might be interested in reducing or modifying their use	*"There are lots of people, in my opinion, who don't necessarily feel like changing, like, necessarily. Plus, you know, it's like: 'Well, me, I want to know what I'm doing (..) '." (FG1)*<br>*"I think that just keeping a logbook, just that, would be an option like that might appeal to a certain crowd." (FG2)*<br>*"Cuz I don't necessarily want to cut down on my consumption, cuz I use it for medicinal purposes (…) It looks like, like what we have here… it doesn't cover the full spectrum of, of… cannabis users, in my opinion." (FG3)*<br>*"There are lots of people, I think, who would like to just have an idea of their consumption before making any sort of decision in this regard." (FG3)*
The proposed structure/sequence was received positively	*"Well, I find that it covered well enough what needed to be done. (…) you know, this thing of going at it step by step, I find that it's… it's a good way of being able to really go at it at your pace." (FG1)*<br>*"Personally, I would have thought that it would have been something a lot heavier, but it's light, it's well structured and I find that it's interesting as an app to really… you have access, it's something simple." (FG2)*<br>*"So, I like the fact that it's short enough. But that at each step we still go straight to the point." (FG2)*
Examples of messages were interesting and well-perceived, but the fine phrasing got mixed reviews	*"I also really liked the situation scenario aspect (…) I think it's a good thing to include situation scenarios like that (…) So I think it's important that it be developed in … the aspect, I mean, in… really concrete situations." (FG2)*<br>*"You know, it seems like it's like too obvious almost (…) it infantilizes the user a lot, I find. Plus I have a feeling that there are a lot of people who will… want to get off it really and who will like… lose interest in using the app (…) Could we not instead find… coping mechanisms that are more optimal, that are more positive, you know (…), I find that it doesn't stick close enough to the reality of users." (FG3)*<br>*"But, I have to admit that the part where, like, when you're hanging out with friends who use or whatever, you know, that, also, I think that it's (…) stuff that people already know (…) it comes across a little like a message from your government, you know. That's what it makes me think of. Messages from your government, they're things that are like a little obvious (…) it's not a bad thing in that, in the end, the fact remains that, yes, it is important that people … well, important… I think that it could reach some people, but I think that there's also another category of people who could like roll their eyes a little when they see messages like that." (FG3)*

Personalized feedback could contribute to reassuring and normalizing their behavior	*"The visual feedback, that's incredible, really. It's really gonna help to … to realize, to become aware. So, yeah, I really like that block there a lot, I find that it's something really good." (FG5)*<br>*"Well, yeah, I find that it's a good thing. Especially to … a little to give advice depending on a person's situation, personally." (FG4)*<br>*"I find it's a good thing that it's personalized because, you know, sometimes there are lots of apps these days or, well, like government websites that it's just like information that's so general that sometimes it doesn't really apply to you." (FG3)*
